# Miniature Rifle Shooting

**Published:** 1908-02-01

**Authors:** 


### The Practitioner's Relaxations and Hobbies.

### MINIATURE RIFLE SHOOTING.

The doctor's work is of such a nature that he has difficulty in finding a recreation that will not interfere too much with his work. A round of golf will occupy three hours at least: a day's shooting or fishing require him to be still more prodigal of time, and, rightly or wrongly, patients are never satisfied unless their doctor is always "on the spot" and within call when needed. To miniature rifle shooting none of these objections can be urged. Inside or in his garden, winter or summer, the doctor can ply this sport and be ever at the service of his patients. The outlay is small. Ammunition suitable for indoor or garden use is cheap, and, after a rifle and target, that is the only expense incurred.

The rifle required is of .22 calibre, of which there are endless makes on the market. For target use the British War Office miniature rifle of the improved pattern is the best. In the first issue the extractor was liable to blow out, but that defect is now remedied. For those who wish to extend their shooting to rooks and rabbits, a repeating weapon, such as the Winchester one sees at fairs, circuses, etc., is more suitable, affording a second, a third, or a twelfth shot if desired, though the sighting of this rifle sometimes requires improvement at the hands of a good gunmaker. Another weapon largely used by miniature shots is the soundly built "Greener," a very reliable rifle of Martini pattern, but somewhat more expensive than those first mentioned.

Those thinking of taking up this hobby should provide themselves with a patent target. A good one catches all the lead, prevents any rebound, and secures persons and furniture from any danger or damage. Moreover, the lead collected in them will pay for the target several times over in a good year's shooting. With suitable ammunition the range may be anything from 15 feet to 25 yards. For ranges above this a heavier cartridge should be employed, as a slight breeze is sufficient to deflect the light bullet. Such a cartridge is the C.B. cap, which costs a shilling a hundred rounds. Smoke and noise are practically non-existent, and the powder charge is wonderfully uniform considering the small quantity in each.

Having provided himself with these necessaries the doctor sallies out to his garden after lunch, let us say, for a shot or two before his consulting hour. His target is at the end of his garden; he himself lies at the other on cushions or a mat with rifle, cartridges, and a field-glass to note his shots. "Very dull," you say. Some day one's wife comes out, and is jocularly offered a shot, for are not all martial instincts confined to males? Madame will not lie down: creations would be crushed: a second's deliberation, a report, and a—no, surely not a bull. The glass is hazy. You examine the card. Yes, undoubtedly, you admit grudgingly the success of madame. Your dullness vanishes. You seize the rifle, but the bull has become elusive—harder to touch than a 6-foot putt. The die is cast, and you are a keen rifleman. Many half-hours slip pleasantly away in this fashion. In time one's aim is seven or ten successive bull's-eyes, or how many successive shots one can put into a calling card. One man we know is trying to put more than four successive shots into a postage stamp at 15 paces. A friend comes and laughs perchance. It looks so simple. He will try. He must have been smoking too much lately, he thinks. Give madame a shot. So you make keen riflemen.

